# Supplementary material for: Systematic identification of non-coding pharmacogenomic landscape in cancer
Source: Nat Commun. 2018 Aug 9;9:3192. doi: 10.1038/s41467-018-05495-9 (PMC6085336; doi:10.1038/s41467-018-05495-9)
Supplement: Supplementary file 1 — Supplementary Information [file 41467_2018_5495_MOESM1_ESM.pdf]

Systematic Identification of Non-coding Pharmacogenomic Landscape in Cancer

Wang *et. al.*

Supplementary Figure 1

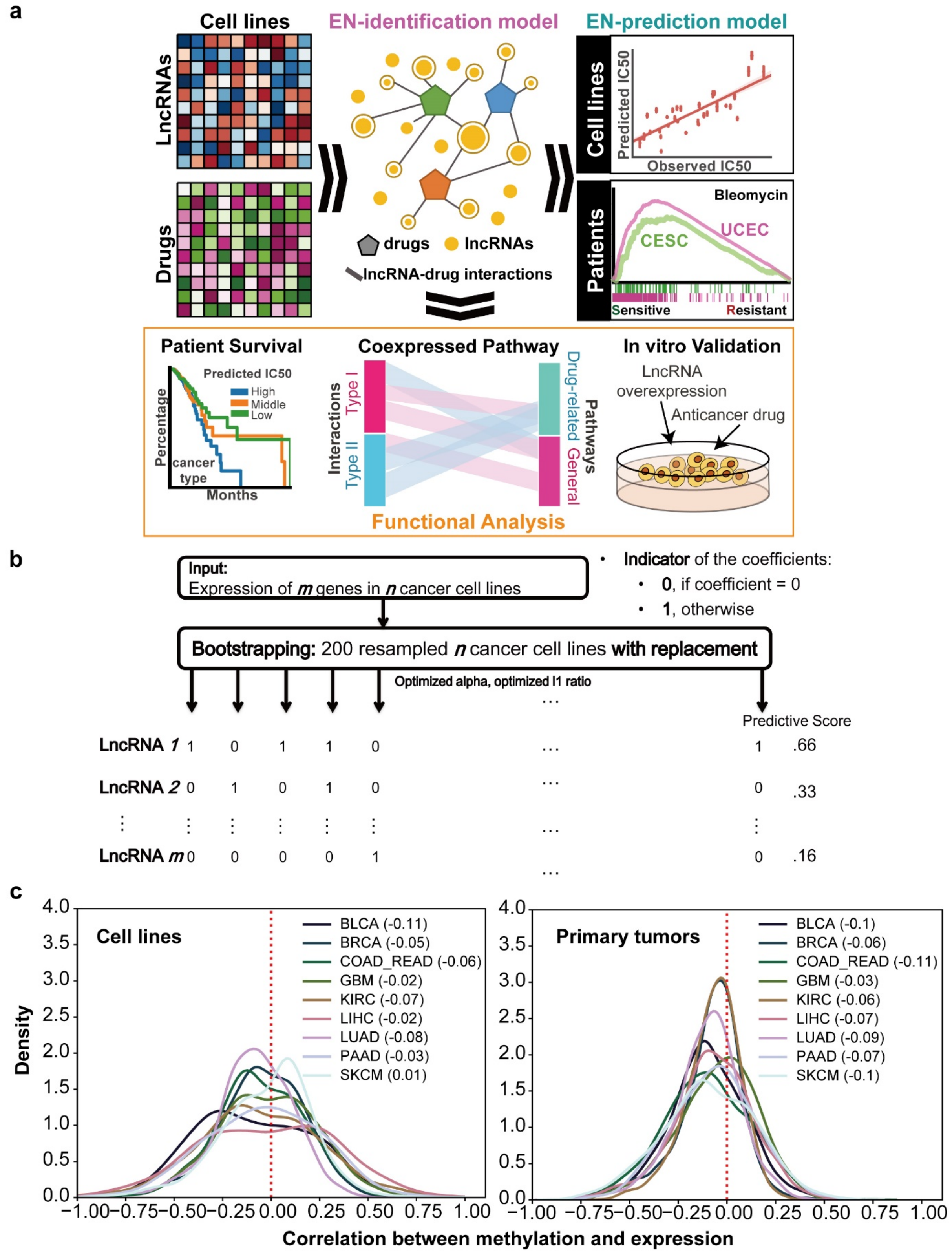

Supplementary Fig.1. Overview of the drug response model. **Related to Fig. 1. (a)** A flow chart of building lncRNA-based EN models. **(b)** The bootstrapping procedure and the calculation of predictive

score. **(c)** Correlation between lncRNA expression and methylation in cancer cell lines and primary tumors. The median of the correlation coefficient for each cancer type is shown in the close parenthesis.

Supplementary Figure 2

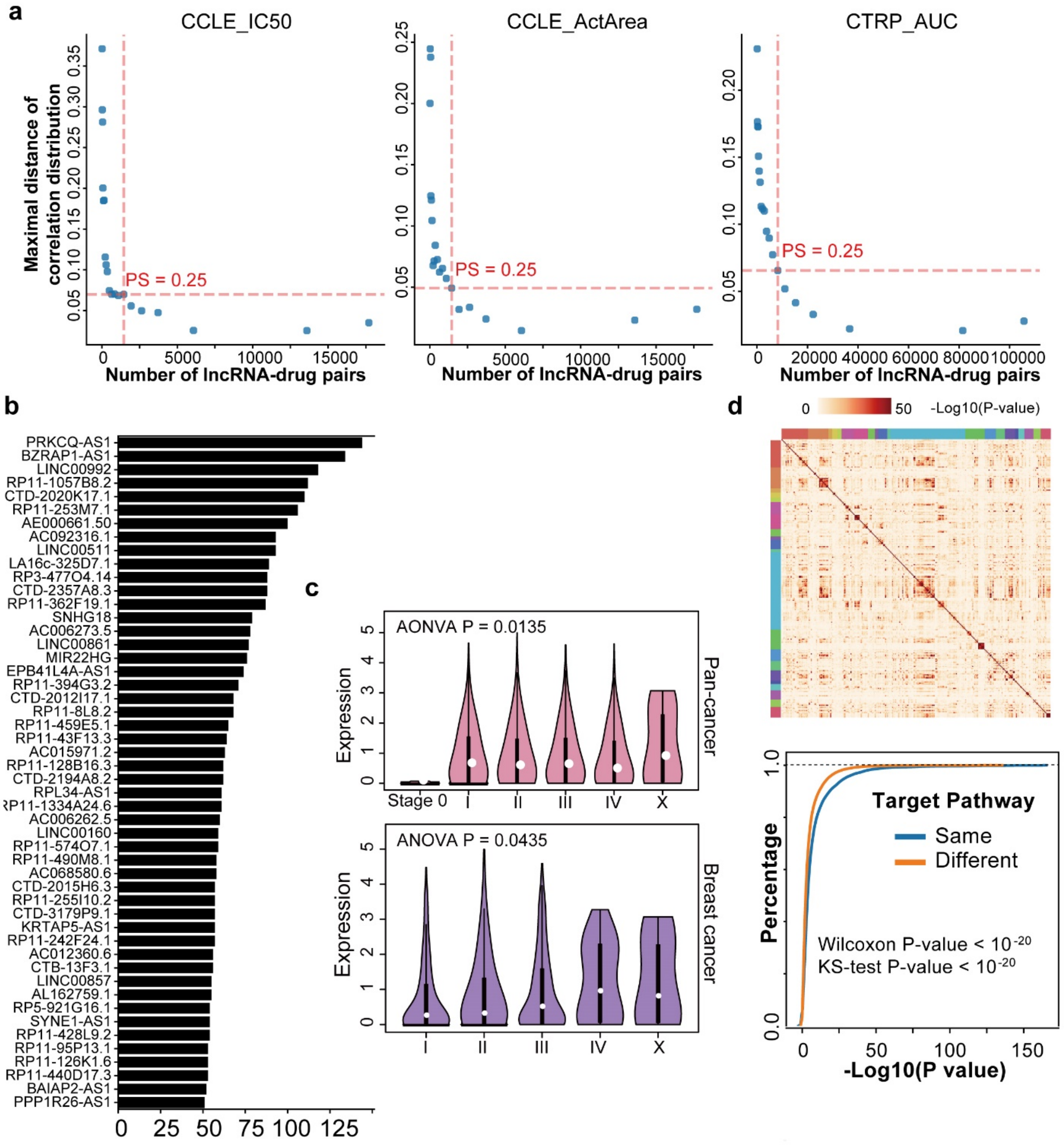

Supplementary Fig.2. A Landscape of LncRNA-Drug Interactions in Cancer Cell Lines. Related to Fig. 2. **(a)** Differences between Spearman's correlation distribution of predictive and non-predictive lncRNA-drug pairs in independent databases CCLE (left and middle) and CTRP (right). X-axis shows the number of lncRNA-drug pairs at the corresponding cutoff. Y-axis shows the maximal distance between two cumulative distributions of correlation coefficients. **(b)** Number of agents to which each lncRNA is selected as a

predictor. **(c)** *LINC00992* expression across TCGA cancer tumors with different stages. **(d)** Similarity between predictive lncRNA selected by different agents. The upper heatmap is clustered based on the agent category using average linkage and Euclidean distance. The colors on the top and the left indicate the categories of agents. The color map is the same as the volcano plot in **Fig.2c**. The below cumulative distribution of two-tailed Fisher's exact test p-value shows the similarity between predictive lncRNA selected by different agents.

Supplementary Figure 3

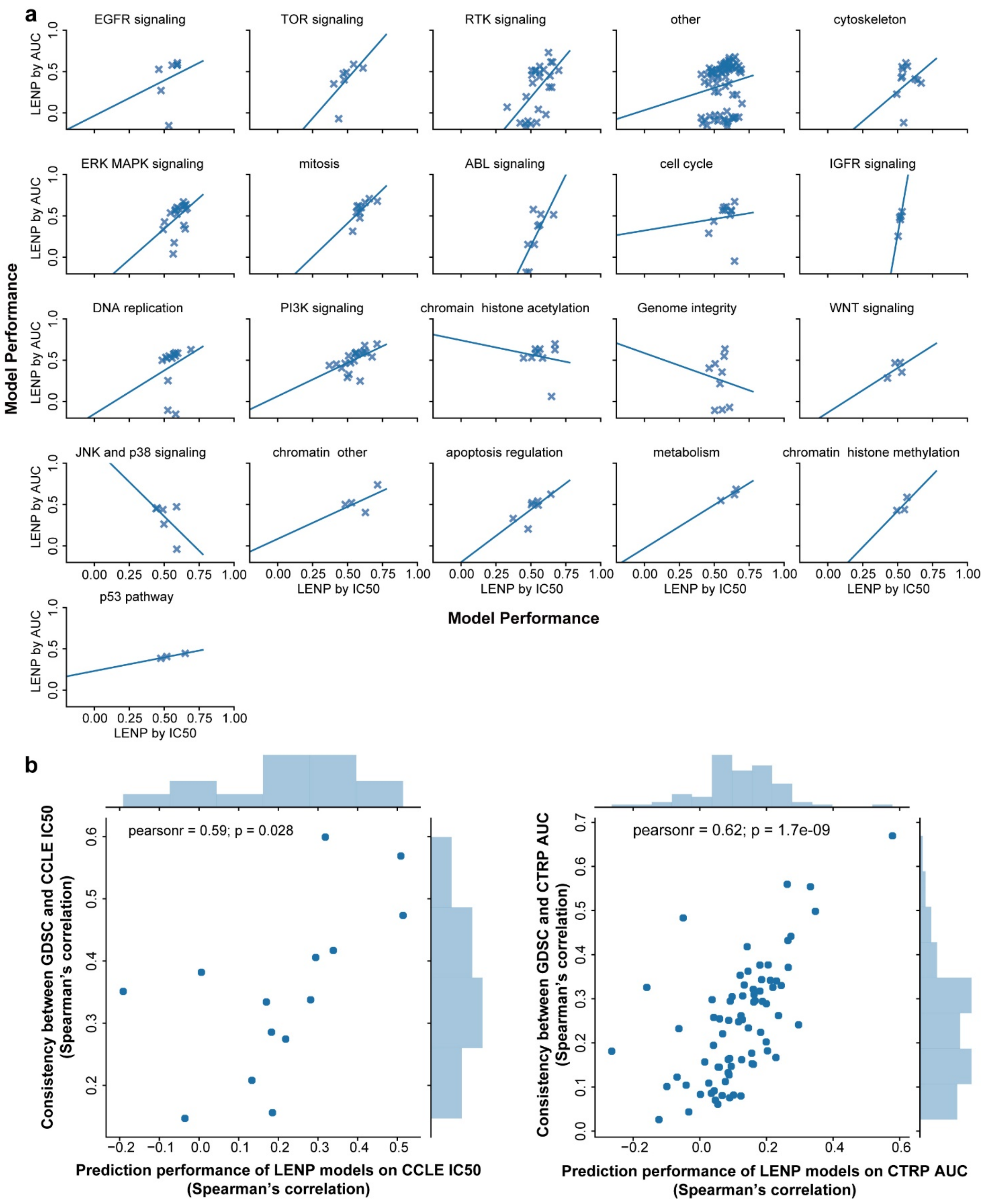

Supplementary Fig.3. Prediction performance of lncRNA-based EN models (LENP), Related to Fig. 3.

(a) Comparison of model performance between LENP training by AUC (y-axis) and LENP training by IC50s

(x-axis) within agent categories. Each cross marker represents one agent. A regression line is drawn for each comparison. **(b)** Correlation between LENP prediction performance and inter-database consistency in CCLE (left) and CTRP (right).

Supplementary Figure 4

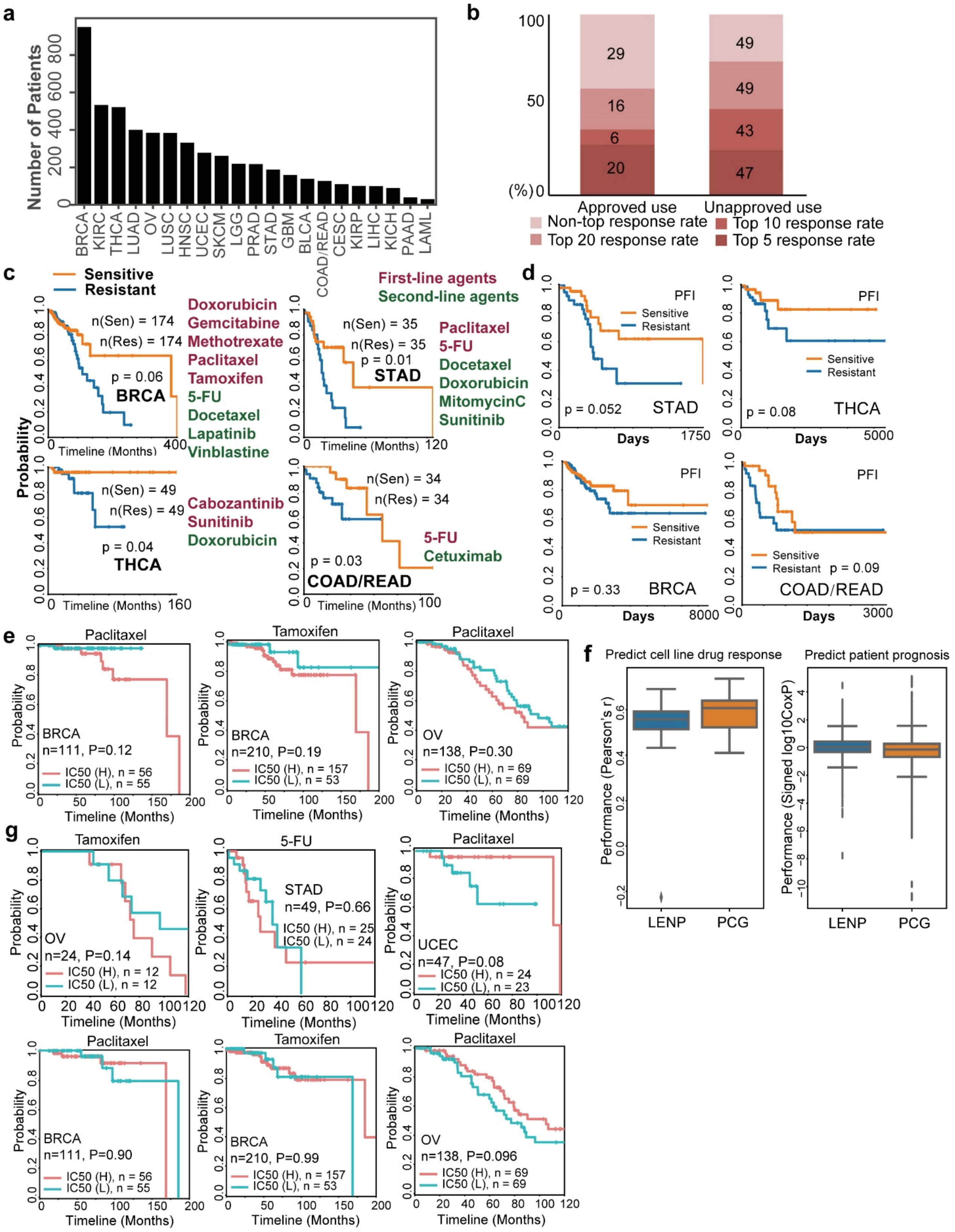

Supplementary Fig.4. Prediction of Drug Response in Cancer Patients Using LncRNA-based EN Models (LENP), Related to Fig. 4. (a) The number of patients with non-stage-I disease (except for LAML)

in each cancer type. **(b)** The proportion of predicted patient response rate in FDA approved indication (i.e., a drug has approved to treat a specific cancer type by FDA) and indication not being approved yet. **(c)** The Kaplan-Meier curves of overall survival for patients grouped by different predicted responses to FDA-approved first- and second-line cancer drugs in four cancer types. **(d)** Kaplan-Meier plot progression free interval (PFI) of patients grouped by weighted-rank predicted drug response in four cancer types. The p-values indicate the significance given by log-rank test. **(e)** The Kaplan-Meier curves of overall survival for OV, STAD and UCEC patients segregated by median predicted IC50s of the received treatments. The IC50s are predicted by LENP models. **(f)** Performance of predicting drug response in cell lines (left) and prognosis in patient (right) by LENPs and PCG-based models. **(g)** The Kaplan-Meier curves of overall survival for BRCA, OV, STAD and UCEC patients segregated by median predicted IC50s of the received treatments. The IC50s are predicted by PCG-based models.

Supplementary Figure 5

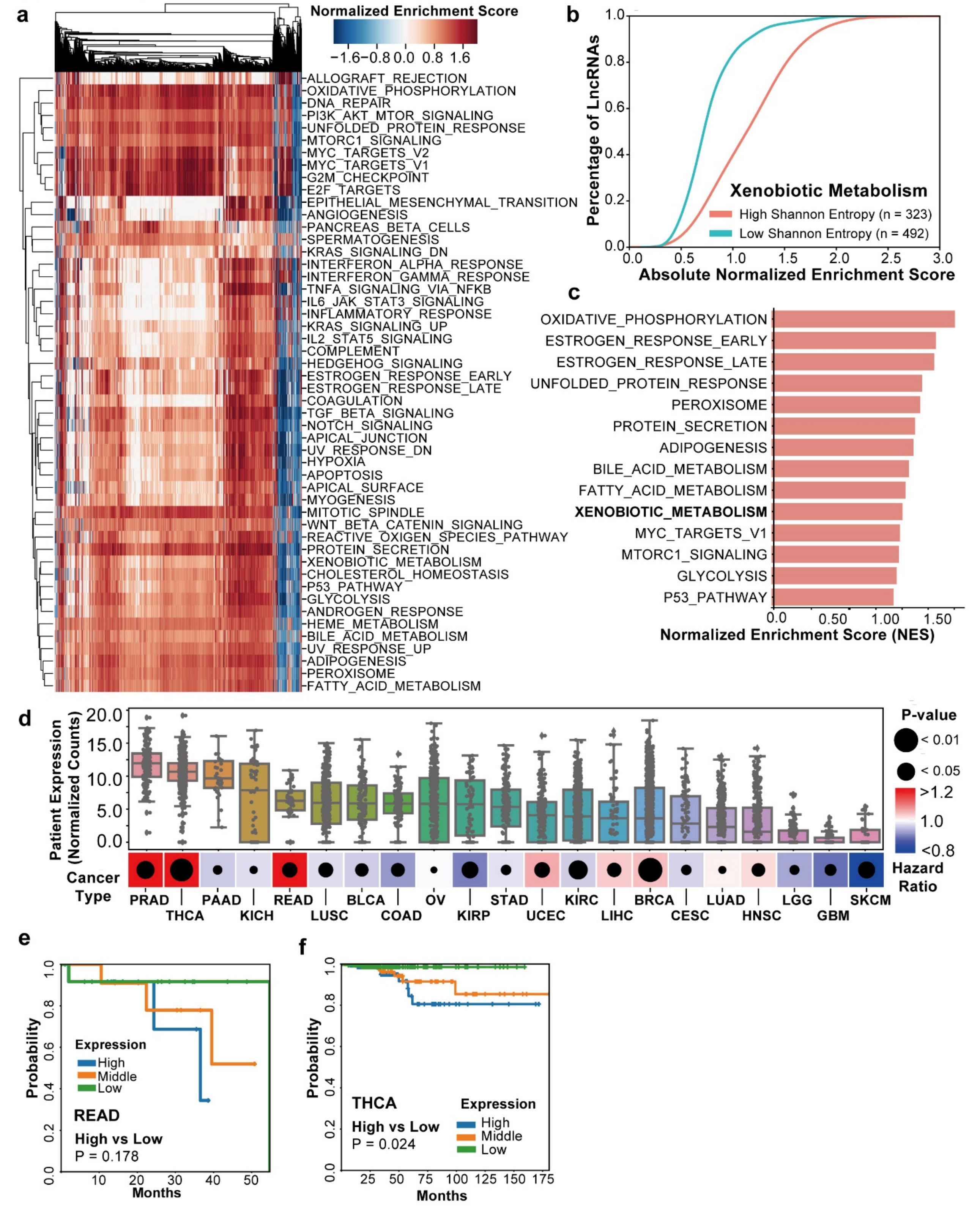

Supplementary Fig.5. Gene Set Enrichment Analysis of LncRNAs Expression across Cell Lines, Related to Fig. 5. **(a)** Heatmap of normalized enrichment score (NES) in gene set enrichment analysis (GSEA) for top predictive lncRNAs. Red (blue) denotes positive (negative) nominal enrichment in Hallmarks. **(b)** Cumulative

distribution of absolute GSEA NES score in lncRNAs with high (low) Shannon entropy. Red (blue) denotes lncRNAs that have high (low) level of entropy. **(c)** Top *LINC00992* associated pathways across cancer cell lines (FDR <= 0.25). The height of the bar indicates the NES score in corresponding pathways. **(d)** The expression of *LINC00992* in cancer patients and its association with patient survival. The upper boxplot indicates the expression (normalized counts) of *LINC00992* in 21 cancer types. The lower heatmap indicates the hazard ratio given by univariate cox regression. The red (blue) indicates a positive (negative) hazard ratio. The size of the inner circle denotes the significance of hazard ratio. **(e)** and **(f)** The Kaplan-Meier curves of overall survival for patients grouped by *LINC00992* expression level in READ **(e)** and THCA **(f)**.

Supplementary Figure 6

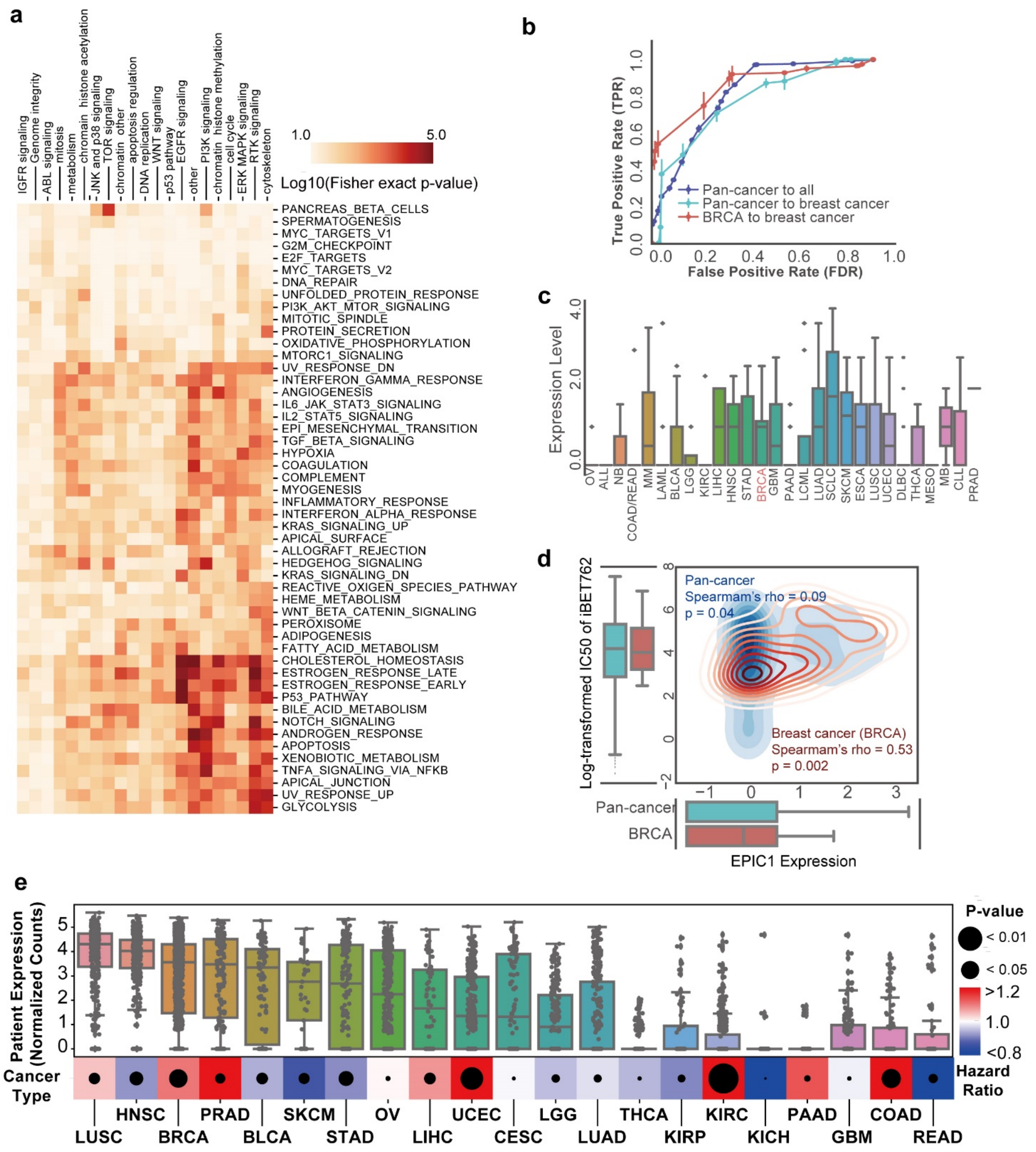

Supplementary Fig.6. Predictive LncRNA Case Study: Expression of *EPIC1* in Different Cancer Types and its Roles in Drug Resistance, Related to Fig. 6. (a) Enrichment of significant lncRNA-pathway association (Bonferroni corrected $p < 0.05$) picked by agents from different target pathways. The color in each cell indicates the significance of enrichment by negative log-transformed p value from Fisher exact test. **(b)** ROC curves with standard error bars of three prediction models listed in the legend for I-BET-762. **(c)** *EPIC1*

expression across cell lines grouped by cancer types. **(d)** Joint-density plot showing the correlation between *EPIC1* expression and IC50 of iBET762 in pancan cell lines. The y-axis and the box plot on the left show the minus ln-transformed IC50 of iBET762 in pancan cell lines (blue) and the breast cancer cell lines (red). The x-axis and the box plot on the bottom show the log-transformed expression of *EPIC1* in all of the cancer cell lines (blue) and the breast cancer cell lines (red). **(e)** The expression of *EPIC1* in cancer patients and its association with patient survival. The upper boxplot indicates the expression (normalized counts) of *EPIC1* in 21 cancer types. The lower heatmap indicates the hazard ratio given by univariate cox regression. The red (blue) indicates a positive (negative) hazard ratio. The size of the inner circle denotes the significance of hazard ratio.

Supplementary Figure 7

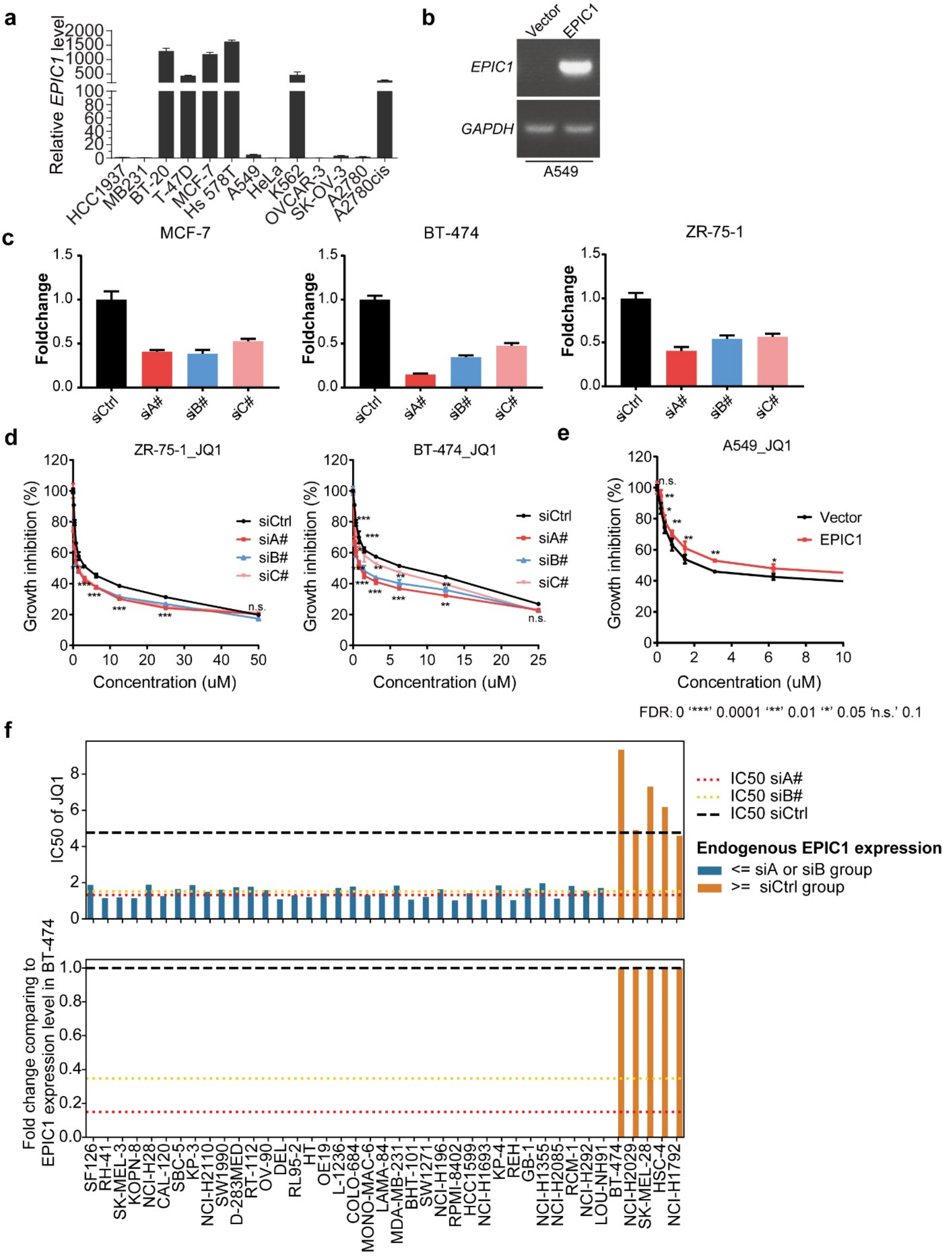

Supplementary Fig.7. Knockdown and overexpression of *EPIC1* in Breast Cancer Cell Lines and its Roles in iBETs Resistance, Related to Fig. 6. (a) Relative expression level of *EPIC1* in 13 cell lines tested

by q-rtPCR. **(b)** The *EPIC1* overexpression in A549 cancer cell. **(c)** the knockdown efficiency of *EPIC1* by three siRNAs in MCF-7, BT-474, and ZR751. **(d)** Growth inhibition curves for *EPIC1* knockdown or control ZR751 (left) and BT474 cells (right) treated with BET inhibitor JQ-1. **(e)** Growth inhibition curves for *EPIC1* overexpressed or control A549 cells treated with BET inhibitor JQ-1. **(f)** Comparison of IC50s between the cells with similar endogenous expression level as *EPIC1* knockdown in BT474. The upper bar plot shows the IC50s of JQ1 across cell lines in GDSC database. The dash lines indicate the IC50 levels after siEPIC1 (yellow and red) or siControl (black) treatments. The lower bar plot shows the endogenous expression levels of *EPIC1* (normalized to *EPIC1* expression in BT474) in the corresponding cell lines. The dash lines indicate the *EPIC1* expression levels after siEPIC1 (yellow and red) or siControl (black) treatments in BT-474 cells.

Supplementary Figure 8

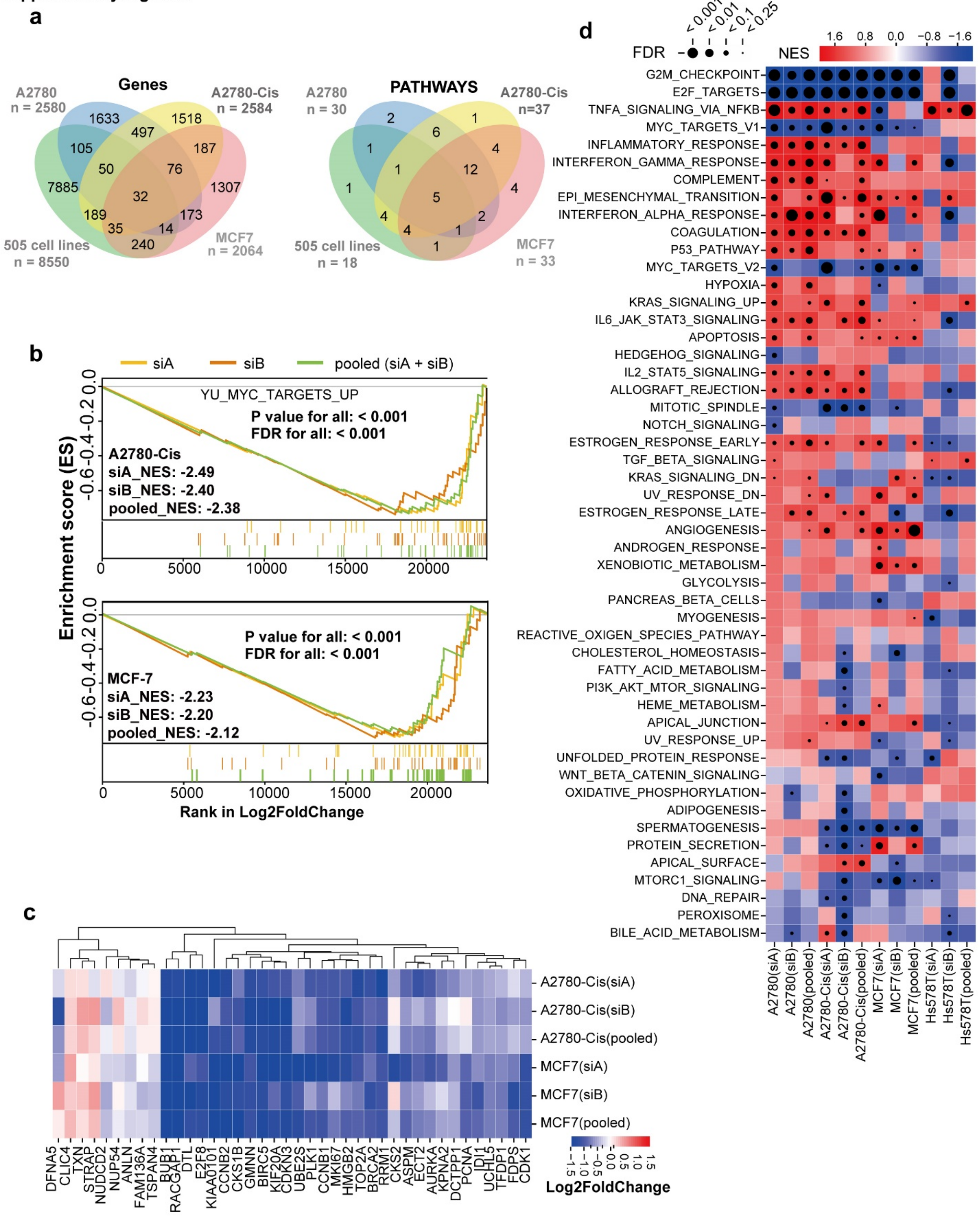

Supplementary Fig.8. RNA-seq Analysis of *EPIC1*'s Function in Breast Cancer and Ovarian Cancer Cell Lines, Related to Fig. 6. (a) Overlapped *EPIC1*-regulated genes/pathways between knockdown cell lines in RNA-seq analysis and 505 cell lines from GDSC. **(b)** Down regulation of cMYC-targets in *EPIC1*

knockdown A2780-Cis and MCF-7 cell lines. **(c)** Expression alteration of cancer hallmark pathways in *EPIC1* knockdown cell lines. The red (blue) indicates an up (down) regulation. The size of the inner circle indicates the false discovery rate. **(d)** Expression alteration of cMYC-targets in *EPIC1* knockdown cell lines. The red (blue) indicates an up (down) regulation.
