# Supplementary material for: Systematic identification of non-coding pharmacogenomic landscape in cancer
Source: Nat Commun. 2018 Aug 9;9:3192. doi: 10.1038/s41467-018-05495-9 (PMC6085336; doi:10.1038/s41467-018-05495-9)
Supplement: Supplementary file 3 — Description of Additional Supplementary Files [file 41467_2018_5495_MOESM3_ESM.pdf]

Description of Additional Supplementary Files

File Name: **Supplementary Data 1**
Description: **The concordance of lncRNAs alterations between cell Lines and primary tumors**. This table provide the original pairwise correlation matrix of lncRNA expression, methylation, copy number alteration and merged profiles for nearest neighbor matching.

File Name: **Supplementary Data 2**
Description: **Drug response data used in this study.** These data provide a list of target information and drug response of 265 GDSC agents analyzed in this study. The overlap agents among CCLE, CTRP and GDSC are also included in this data.

File Name: **Supplementary Data 3**
Description: **The landscape of lncRNA-drug predictive pairs.** These data provide the list of pan-cancer and cancer-specific lncRNA-drug predictive pairs identified by elastic net regression and bootstrapping procedure.

File Name: **Supplementary Data 4**
Description: **Elastic-Net regression model training and validation**
These tables provide the final model performance of pan-cancer and cancer specific models using Pearson's correlation and Kendall's tau.

File Name: **Supplementary Data 5**
Description: **Prediction of patient drug response by LENPs.** This data proved the predicted IC50s and response rate of FDA approved agents in patients (stage >= II except for LAML) by lncRNA-based EN pan-cancer models. The information of first line and second line therapeutics recorded in National Comprehensive Cancer Network (NCCN) guidelines (www.nccn.org) is also included in this data.

File Name: **Supplementary Data 6**
Description: **Gene Sets Enrichment Analysis of Predictive LncRNAs**
This data provide the list of multi-drug-response (MDR) related lncRNAs identified by entropy approach and the correlation between Shannon entropy and NES of lncRNAs in cancer hallmarks.

File Name: **Supplementary Data 7**
Description: **List of xenobiotic metabolism related lncRNAs**. This data provide the list of MDR-related lncRNAs that significantly associate with xenobiotic metabolism, the association of *LINC00992* with cancer hallmarks in cell lines, and a list of agents that select *LINC00992* as top predictor of drug response.

File Name: **Supplementary Data 8**
Description: **Gene Sets Enrichment Analysis of Predictive LncRNAs**. This data provide the matrix of significant association between predictive lncRNAs and cancer hallmarks.

File Name: **Supplementary Data 9**
Description: **Role of *EPIC1* in inducing cell lines' resistance to BET inhibitors**. This data provided the information of EPIC1 expression (normalized counts) across cancer cell lines in this study and the *EPIC1*'s predictive scores in BRCA-specific LENP models.
